# The Impact of Intermittent Fasting (Ramadan Fasting) on Psoriatic Arthritis Disease Activity, Enthesitis, and Dactylitis: A Multicentre Study

**DOI:** 10.3390/nu11030601

**Published:** 2019-03-12

**Authors:** Mohammad Adawi, Giovanni Damiani, Nicola Luigi Bragazzi, Charlie Bridgewood, Alessia Pacifico, Rosalynn R. Z. Conic, Aldo Morrone, Piergiorgio Malagoli, Paolo Daniele Maria Pigatto, Howard Amital, Dennis McGonagle, Abdulla Watad

**Affiliations:** 1 Padeh and Ziv Hospitals, Azrieli Faculty of Medicine, Bar-Ilan University, 5290002 Ramat Gan, Israel; adawimo1802@gmail.com; 2 Clinical Dermatology, IRCCS Istituto Ortopedico Galeazzi, Department of Biomedical, Surgical and Dental Sciences, University of Milan, 20161 Milan, Italy; dr.giovanni.damiani@gmail.com (G.D.); paolopigatto@valeo.it (P.D.M.P.); 3 Young Dermatologists Italian Network (YDIN), GISED, 24122 Bergamo, Italy; 4 Department of Dermatology, Case Western Reserve University, Cleveland, OH 44124, USA; ruzica.conic@gmail.com; 5 Postgraduate School of Public Health, Department of Health Sciences (DISSAL), University of Genoa, 16132 Genoa, Italy; 6 NIHR Leeds Biomedical Research Centre, Leeds Teaching Hospitals NHS Trust; Leeds Institute of Rheumatic and Musculoskeletal Medicine, University of Leeds, LS7 4SA Leeds, UK; C.D.Bridgewood@leeds.ac.uk (C.B.); D.G.McGonagle@leeds.ac.uk (D.M.); 7 Clinical Dermatology Department, S. Gallicano Dermatological Institute, IRCCS, 00144 Rome, Italy; alessia.pacifico@tiscali.it (A.P.); aldomorrone54@gmail.com (A.M.); 8 Dermatology Unit, Azienda Ospedaliera San Donato Milanese, 20097 Milan, Italy; dermapier@gmail.com; 9 Department of Medicine ‘B’, Sheba Medical Center, Tel-Hashomer and Sackler Faculty of Medicine, Tel Aviv University, 5265601 Tel Aviv, Israel; Howard.Amital@sheba.health.gov.il

**Keywords:** psoriatic arthritis, intermittent fasting, circadian rhythm and biological clock, chronomedicine

## Abstract

Intermittent circadian fasting, namely Ramadan, is a common worldwide practice. Such fasting has a positive impact on psoriasis, but no data exist on its role in psoriatic arthritis (PsA)—a disease that is clearly linked to body mass index. We enrolled 37 patients (23 females and 14 males) with a mean age 43.32 ± 7.81 and they fasted for 17 h for one month in 2016. The baseline PsA characteristics were collected and 12 (32.4%) patients had peripheral arthritis, 13 (35.1%) had axial involvement, 24 (64.9%) had enthesitis, and 13 (35.1%) had dactylitis. Three patients (8.1%) were treated with methotrexate, 28 (75.7%) with TNF-α blockers, and 6 (16.2%) with IL-17 blockers. After a month of intermittent fasting, C-reactive protein (CRP) levels decreased from 14.08 ± 4.65 to 12.16 ± 4.46 (*p* < 0.0001), Bath Ankylosing Spondylitis Disease Activity Index (BASDAI) decreased from 2.83 ± 1.03 to 2.08 ± 0.67 (*p* = 0.0078), Psoriasis Area Severity Index (PASI) decreased from 7.46 ± 2.43 to 5.86 ± 2.37 (*p* < 0.0001), and Disease Activity index for PSoriatic Arthritis (DAPSA) decreased from 28.11 ± 4.51 to 25.76 ± 4.48 (*p* < 0.0001). Similarly, enthesitis improved after fasting, with Leeds Enthesitis Index (LEI) decreasing from 2.25 ± 1.11 to 1.71 ± 0.86 (*p* < 0.0001) and dactylitis severity score (DSS) decreasing from 9.92 ± 2.93 to 8.54 ± 2.79 (*p* = 0.0001). Fasting was found to be a predictor of a decrease in PsA disease activity scores (DAPSA, BASDAI, LEI, DSS) even after adjustment for weight loss. IL-17 therapy was found to be an independent predictor of decreases in LEI after fasting. These preliminary data may support the use of chronomedicine in the context of rheumatic diseases, namely PsA. Further studies are needed to support our findings.

## 1. Introduction

Psoriatic arthritis (PsA) is an inflammatory arthropathy that occurs in up to 30% of people with psoriasis [1]. PsA is considered to be a member of the spondyloarthritis (SpA) family, whose members share several pathogenic and therapeutic aspects [2]. The pathogenesis of PsA is not fully understood, although it is believed to be a result of a complex interplay between genetic background and different environmental factors such as mechanical stress or dysbiosis [3].

Several studies of different designs have shown that PsA has a serious impact on quality of life and can be a very destructive and progressive disease [4]. Moreover, it has been found that patients with PsA have a higher rate of comorbidities, such as cardiovascular diseases, that may affect mortality rates in these patients [5]. Similar to the SpA spectrum of disorders, enthesitis is a cardinal manifestation of PsA and has been associated with worse prognostic outcomes of PsA disease [6,7]. Furthermore, dactylitis is a sometimes disabling PsA-associated manifestation [8]. The introduction of biologic therapy has revolutionized the management of PsA patients in the clinic. However, other factors, including those related to diet (such as weight loss and surgical bypass procedures for obesity), may have a benefit in treating and preventing PsA development.

Ramadan fasting is a religious duty, even though some population demographics such as children, pregnant or breastfeeding women, travelers, and patients are waived. The fasting in Ramadan consists of intermittent fasting that is observed from sunrise to sunset and is alternated with moments of re-feeding [9]. Differently from other fasting and dieting regimens, such as caloric restriction, the Ramadan fasting follows the circadian rhythm. However, despite this peculiarity, the potential impact of intermittent fasting on the immune system has been relatively overlooked.

We have previously published a systematic review that has demonstrated contrasting findings among different studies, although a number of studies showed that intermittent fasting can decrease the levels of several proinflammatory cytokines levels such as IL-1, TNF, and IL-6 [9]. Other studies found that fasting can impact on neutrophils function by attenuating the release of myeloperoxidase enzyme, reduction of the production of leukotrienes, and altering the fatty acid composition of membrane phospholipids leading to the decrease of inflammatory markers and symptoms [10]. There is very little data concerning the link of fasting and the activity of specific autoimmune diseases. Recently we showed beneficial effects of Ramadan fasting on the activity of psoriasis diseases expressed in the Psoriasis Area Severity Index (PASI) [11]. However, there are no studies assessing the impact of fasting on PsA disease activity or related disorders including enthesitis and dactylitis. Therefore, this study aimed to examine prospectively the effect of intermittent fasting during Ramadan on PsA disease activity, enthesitis and dactylitis.

## 2. Materials and Methods

### 2.1. Ethical Approval

The research received ethical approval from the ethical committees of the three medical centers involved with this study (the San Gallicano’s Dermatological Institute, Rome, Italy; the Dermatology Unit of the hospital IRCCS Istituto Ortopedico Galeazzi, Milan, Italy; and the Dermatology Unit of the hospital IRCCS San Donato, Milan, Italy) and was carried out according to the 1964 Helsinki’s declaration and its subsequent amendments (sub-analysis of the study protocol ID T-ART, date of approval 17 July 2014, amended on 10 July 2015). Written informed consent was obtained from each participant in the study.

### 2.2. Patients’ Selection and Inclusion

Patients were included in the study from three medical centers (the San Gallicano’s Dermatological Institute, Rome, Italy; the Dermatology Unit of the hospital IRCCS Istituto Ortopedico Galeazzi, Milan, Italy; and the Dermatology Unit of the hospital IRCCS San Donato, Milan, Italy). Inclusion criteria were as follows: (i) age greater than 18 years; (ii) being diagnosed with PsA according to the Classification for Psoriatic Arthritis (CASPAR) criteria; (iii) planning to fast the whole month of Ramadan; (iv) no significant difference in the severity of PsA disease among the last two clinical visits (<10% of change in severity score); v) all the current medications were in the maintenance phase of administration and not induction phase. Exclusion criteria were defined as the following: (i) pregnancy or breast feeding; (ii) seropositivity for hepatitis C virus (HCV), hepatitis B virus (HBV), or human immunodeficiency virus (HIV); (iii) any medical issue preventing the patient from following the fasting.

### 2.3. Measures and Outcomes

Different biochemical parameters (namely, white blood cells, neutrophils, lymphocytes, red blood cells, mean corpuscular volume, hemoglobin, platelets, creatinine, urea, aspartate aminotransferase, alanine aminotransferase, gamma-glutamyl transferase, alkaline phosphatase, and C-reactive protein or CRP) were collected before and after the Ramadan fasting.

To assess the PsA disease activity, Disease Activity index for PSoriatic Arthritis (DAPSA) was used. This score includes the assessment of tender joints, swollen joints, CRP levels, the activity of the disease and the level of pain, with a score of 0–4 for remission, 5–14 low, 15–28 moderate, >28 high disease activity.

For those with axial involvement detected by spine magnetic resonance imaging (MRI), the Bath Ankylosing Spondylitis Disease Activity Index (BASDAI) was used to assess any change after fasting. The score consists of a 0–10 scale measuring discomfort, pain, and fatigue.

Enthesitis severity was assessed by the Leeds Enthesitis Index (LEI), which consists of the examination of six sites: two sites at each of the lateral epicondyles of the humerus, medial condyles of the femur, and the insertion of the Achilles’ tendon, with a maximum score of 6.

Dactylitis assessment was done by dactylitis severity score (DSS), which is determined by a scale of 0–3 for each digit of the hands and feet, with a total score for a patient ranging from 0 to 60.

DAPSA, BASDAI, LEI, and DSS were evaluated by two different independent board-certified rheumatologists and the average number of each tool was obtained. A third rheumatologist was consulted in cases where there was a significant difference in the computed scores between the two rheumatologists (greater than 5%). Severity and extent of psoriasis was assessed by Psoriasis Area Severity Index (PASI), which was calculated by a dermatologist, with a maximum score of 72.

All scores were calculated 1–3 days before the beginning of the Ramadan fasting, and 1–3 days after the end of the month of Ramadan.

### 2.4. Statistical Analysis

Before handling or analyzing any data, figures were visually inspected in order to capture any potential outlier. Normality of data distribution was checked by conducting the Shapiro-Wilk’s test. This test was preferred to other tests, given the small sample size.

Data were computed as means ± standard deviations for continuous variables, whereas they were expressed as percentages in the case of categorical parameters.

Student’s *t*-test for paired samples was applied to compute the mean differences before and after the Ramadan fasting.

Pairwise Hodges–Lehmann (H-L) median differences were also computed. Multiple regression analyses (using stepwise and enter approach) were conducted in order to shed light on the independent predictors of changes in the different scores.

All statistical analyses were carried out using the commercial software “Statistical Package for the Social Sciences” (SPSS for Windows, version 24, IBM, Armonk, NY, USA) and all graphs were generated using the commercial software MedCalc Statistical Software version 18.11.3 (MedCalc Software bvba, Ostend, Belgium; https://www.medcalc.org).

For all analyses, figures with a *p*-value less than 0.05 were considered to be statistically significant.

## 3. Results

### 3.1. Study Population

Out of 40 patients, 37 (23 females, 14 males) took part in the present study (from 6 June 2016 to 5 July 2016, with a length of fasting of approximately 17 h) (Table 1).

Three patients were excluded due to medical issues that prevented them from continuing fasting (one had pneumonia and was referred to internal medicine, one had gastroenteritis, and the last one had renal failure). The mean age was 43.32 ± 7.81 years (median 43 years) and the mean BMI was 26.16 ± 1.59 kg/m^2^ (median 26 kg/m^2^). Twelve subjects (32.4%) reported a normal weight whereas 25 (67.6%) were overweight.

The PsA disease duration was on average 7.59 ± 3.78 years (median 8 years). Fifteen patients (40.5%) had a positive family history for PsA. All patients were treated with stable dose therapy only, and 3 patients (8.1%) were treated with methotrexate (range 15–20 mg/week), 28 (75.7%) with TNF-α blockers (11 treated with Etanercept 50 mg/week, 11 with Adalimumab 40 mg/week, 3 with Golimumab 50 mg/4 weeks, and 3 with Certolizumab 200 mg/2 weeks), and 6 (16.2%) with IL-17 blockers (4 treated with Secukinumab 150 mg/4 weeks, and 2 with Ixekizumab 80mg/4 weeks).

With regard to PsA disease features at baseline, 12 (32.4%) had peripheral arthritis, 13 (35.1%) had axial involvement, 24 (64.9%) had enthesitis, and 13 (35.1%) had dactylitis.

### 3.2. Impact of Intermittent Fasting on Patient Weight

After the Ramadan fasting, the average weight loss was not different from the one observed before fasting. Twenty-nine patients (78.4%) did not report any change in weight, whereas 4 subjects (10.8%) gained 1 kg and 1 individual (2.7%) gained 2 kg. Three subjects (8.1%) lost 1 kg.

### 3.3. Impact of Intermittent Fasting on Biochemical Parameters

The Ramadan fasting had no impact on any biochemical parameter studied, except for CRP (Table 2).

**Figure 1:**
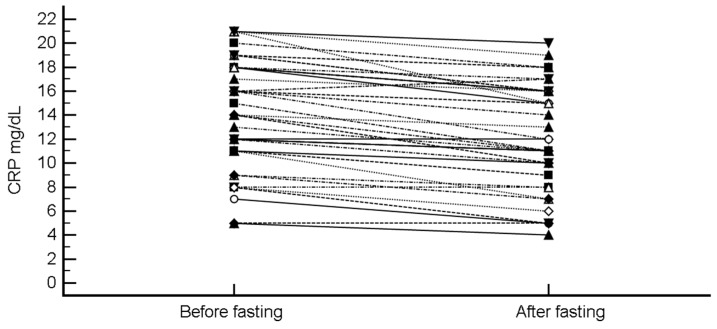
Change in C-reactive protein (CRP) levels after intermittent fasting.

CRP was the only biochemical parameter for which a statistically significant change before and after the Ramadan fasting could be detected. Indeed, CRP levels decreased from 14.08 ± 4.65 to 12.16 ± 4.46 (mean difference −1.92 (95% CI, −2.38 to −1.46), *p* < 0.0001; H-L median difference −2 (95%CI, −2.5 to −1.5), *p* < 0.0001) (Figure 1).

### 3.4. Impact of Intermittent Fasting on PsA Disease Activity Scores, Enthesitis and Dactylitis

**Figure 2:**
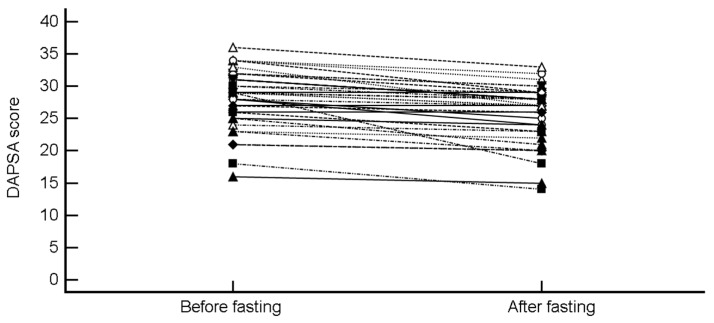
Change in Disease Activity Index for PSoriatic Arthritis (DAPSA) score after intermittent fasting.

Similarly, DAPSA decreased from 28.11 4.51 to 25.76 4.48 (mean difference −2.35 (95% CI, −3.03 to −1.67), *p* < 0.0001; H-L median difference −2 (−2.5 to −1.5), *p* < 0.0001) (Figure 2).

**Figure 3:**
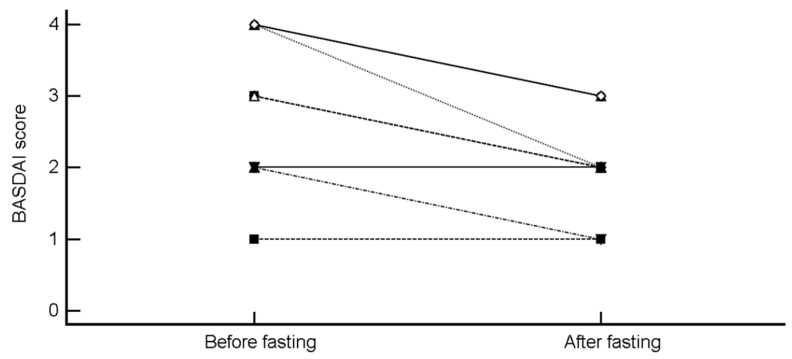
Change in Bath Ankylosing Spondylitis Disease Activity Index (BASDAI) score after intermittent fasting.

BASDAI decreased from 2.83 ± 1.03 to 2.08 ± 0.67 (mean difference −0.76 (95% CI, −1.14 to −0.36), *p* = 0.0015; H-L median difference −1 (95% CI, −1 to −0.5), *p* = 0.0078) (Figure 3).

**Figure 4:**
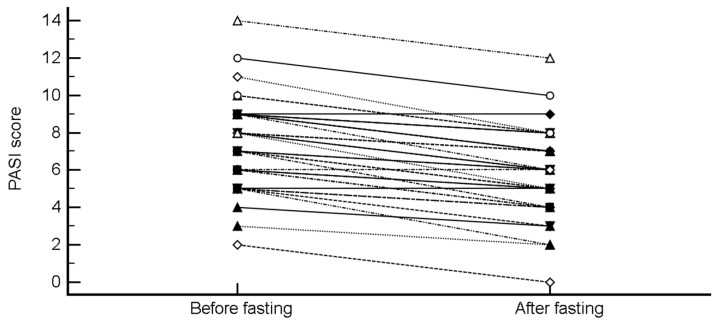
Change in Psoriasis Area Severity Index (PASI) after intermittent fasting.

PASI decreased from 7.46 ± 2.43 to 5.86 ± 2.37 (mean difference −1.59 (95% CI, −1.87 to −1.32), *p* < 0.0001; H-L median difference −1.5 (95% CI, −2 to −1.5), *p* < 0.0001) (Figure 4).

**Figure 5:**
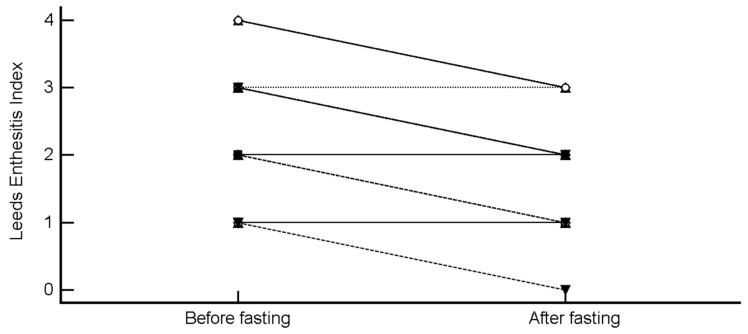
Change in Leeds Enthesitis Index after intermittent fasting.

LEI decreased from 2.25 ± 1.11 to 1.71 ± 0.86 (mean difference −0.54 (95% CI, −0.76 to −0.33), *p* < 0.0001; H-L median difference -0.5 (95% CI, −1 to −0.5), *p* = 0.0002) (Figure 5).

**Figure 6:**
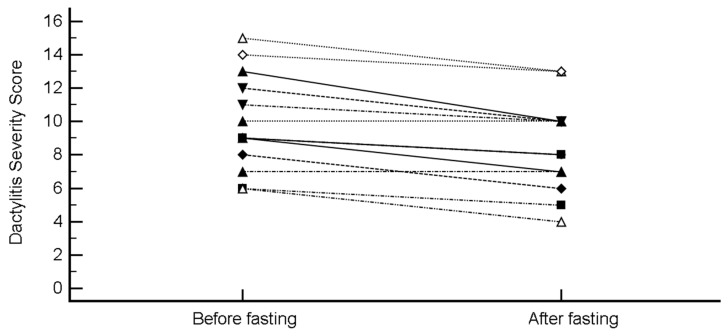
Change in the Dactylitis Severity Score (DSS) after intermittent fasting.

DSS decreased from 9.92 ± 2.93 to 8.54 ± 2.79 (mean difference −1.38 (95% CI, −1.91 to −0.86), *p* = 0.0001; H-L median difference −1.5 (95% CI, −2 to −1), *p* = 0.0010) (Figure 6).

At the multivariate regression analyses (stepwise approach), these improvements occurred regardless of age, gender, BMI, pharmacological treatment, disease duration, clinical symptoms, and family history for PsA.

However, peripheral arthritis was found to be an independent predictor of a prominent decrease of CRP levels after fasting compared to those with axial involvement (regression coefficient = 0.98, standard error (SE) = 0.46, *p* = 0.0410, *r_partial_* = 0.34, *r_semipartial_* = 0.34).

Of note, this also may be related, in that patients with peripheral arthritis tend to have higher levels of CRP than those with axial involvement.

Multivariate regression analyses (enter approach) investigating the main covariates associated with improvements in clinical scores after fasting found no significant independent predictors except for therapy, which was found to be associated with decreases in LEI (Table 3).

These were greater in those treated with IL-17 blockers and lower in those under methotrexate.

## 4. Discussion

This is the first study to demonstrate that intermittent fasting has beneficial effects on PsA disease expressed by DPSAI, BASDAI, enthesitis, and dactylitis. This benefit was not linked to weight loss, which remained stable in the cohort throughout the study. Indeed, even after adjusting for weight change, the benefit of fasting on PsA disease activity scores remained statistically significant.

Dietary habits have been found to have a significant impact on human health, affecting the risk for hypertension and cardiovascular diseases, as well as influencing the risk of developing malignant conditions and impacting on mortality [12]. Nutritional status and dietary compounds have long been recognized as important factors in the alteration of immune system activity. Indeed, salt has been reported to enhance inflammation via the increase of IL-17A levels and reduction of T regulatory function [13]. By contrast, other dietary compounds such as curcumin and capsaicin in spicy foods and resveratrol contained in chocolate are able to attenuate the immune system reactions and activity by increasing M2/M1 macrophages and also B regulatory cells function [13]. Although with inconclusive results, the so-called “Western diet” has been linked with an increased risk of autoimmune diseases such as Crohn’s diseases and ulcerative colitis [14]. On the other hand, higher consumption of extra virgin olive oil was found to be associated with a lower psoriasis severity [15]. This is believed to be attributed to oleocanthal, a phenolic compound contained in extra virgin olive oil that is known to have anti-inflammatory effects. However, very few studies have addressed the effect of fasting subtypes on the immune system activity. An interesting study assessed the impact of a 36-h fasting period followed by a 4-h refeeding period on different components of the immune system in both healthy elderly and young adult volunteers [16]. The study found a significant decrease in the percentage of T-cells subsets in both study populations and this decrease persisted after the refeeding. This may explain some of the beneficial effects induced by fasting in PsA patients, as T-cells are a major player in the disease.

The treatment of PsA can be highly challenging, and very often there is the need for more than one therapeutic agent to control disease. Obesity has been widely linked to psoriasis and PsA [17,18]. Another study has shown that BMI at the age of 18 years but not the current BMI is linked with an increased risk of the development of PsA [19]. It has also been suggested that treatment of obesity may lead to a better management of PsA [20].

However, in our study, fasting led to improvement in PsA disease activity scores, enthesitis, and dactylitis regardless of the change in the patients’ weight, which may support that the fasting process itself can affect the immune system activity. This is not surprising, as it has already been reported that intermittent fasting can induce reduction in the proinflammatory cytokines, increase the function of T regulatory cells, and decrease the activity of Th17 cells, all of which are known to be involved in PsA pathogenesis [9].

In man, circadian rhythms are biological processes that synchronize to the rotation of the Earth or a 24-h period. Numerous events can cause disruptions of the circadian clock, such as jet lag (de-synchrony), light/darkness, as well as night shift working [21,22,23].

Circadian rhythms are involved in the regulation of many activities including sleep, feeding times, energy metabolism, as well as both the endocrine and the immune systems [23]. When the circadian rhythm is altered, such as that caused by sleep and feeding, this has been linked to the development of multiple immunological disorders such as rheumatoid arthritis, cardiovascular disease, asthma, and atopic dermatitis [24,25].

During fasting, such as Ramadan, disturbances and alterations in sleep patterns are commonly observed [26]. However, whether Ramadan causes disturbances to circadian rhythm is heavily debated [27]. The majority of immune cells express circadian clock genes [28]. The circadian rhythm in immune cells is associated with cytokine/chemokine release, cell migration, phagocytosis, and cytolytic activity. Circadian disruption has been extensively studied in animal models, by inducing ‘chronic jet let’ (regular shifts of light), and this was found to induce negative effects on immune cell function and lead to increased mortality [29,30]. There are also multiple research lines implicating circadian rhythm with the closely related psoriasis. Psoriasis is associated with sleep disorders [31], moreover, in a mouse model of psoriasis, circadian clock genes were found to regulate skin inflammation [32].

It also important to mention that during Ramadan, patients tend to administer their drugs during the night and this may have beneficial effects, as several studies have suggested that bedtime or night doses compared with morning administration can lead to a better effect of the drug [33,34,35].

However, our study has several limitations that should be acknowledged. Firstly, our study had a small sample size due to the extreme difficulty of recruiting patients with PsA that fast the whole month and were interested in taking part in the study. One month may be a short period to observe all the potential effects of intermittent fasting on rheumatic disease activity and therefore longer observation time frames are warranted.

A further limitation was the inability to monitor potential dietary habit changes of the individuals, which can significantly impact on the disease control. As fasting is normally followed by significant changes in the daily diet, there may be dietary factors with the potential to impact the PsA disease activity that should be addressed in future studies.

## 5. Conclusions

In conclusion, intermittent fasting was shown to have beneficial effects on PsA disease activity, including PsA-related disorders such as enthesitis and dactylitis, regardless of the implicated drug therapy. These findings may be translated into new management strategies of PsA patients. However, larger studies are needed to replicate our findings, and longer observation time frames (for instance, studying several months of fasting) are recommended.

## Figures and Tables

**Table 1:** Main characteristics of the study population.

Parameter		Value
Age (years)		43.32 ± 7.81 (median 43)
Gender (%)	Male	14 (37.8%)
	Female	23 (62.2%)
BMI (kg/m^2^)		26.16 ± 1.59
Disease duration (years)		7.59 ± 3.78 (median 8)
Family history positive for PsA		15 (40.5%)
Country of origin	Egypt	10 (27.0%)
	Morocco	6 (16.2%)
	Algeria	5 (13.5%)
	Turkey	5 (13.5%)
	Israel	3 (8.1%)
	Kurdistan	3 (8.1%)
	Afghanistan	2 (5.4%)
	Cyprus	2 (5.4%)
	Jordan	1 (2.7%)
Enthesitis		24 (64.9%)
Dactylitis		13 (35.1%)
Axial involvement		13 (35.1%)
Peripheral arthritis		12 (32.4%)
Treatment	Methotrexate	3 (8.1%)
	TNF-α blockers	28 (75.7%)
	IL-17 blockers	6 (16.2%)

Abbreviations: BMI, body mass index; IL-17, interleukin-17; PsA, psoriatic arthritis; TNF-α, tumor necrosis factor alpha.

**Table 2:** Biochemical parameters before and after the Ramadan fasting.

Parameter	Before Fasting	After Fasting
WBC (N/μL)	7850 ± 430	7750 ± 510
Neutrophils (10^9^/L)	5.4 ± 0.8	5.4 ± 0.6
Lymphocytes (10^9^/L)	2.7 ± 0.4	2.8 ± 0.4
RBC (10^6^/μL)	5.6 ± 1.2	5.7 ± 1.1
MCV (fL)	89.8 ± 2.3	90 ± 2.1
Hb (g/dL)	14.8 ± 1.3	14.7 ± 1.4
PLT (1,000/μL)	215 ± 32	216 ± 28
Creatinine (mg/dL)	0.7 ± 0.1	0.7 ± 0.1
Urea (mg/dL)	7.3 ± 0.9	6.9 ± 1.2
AST (IU/L)	37 ± 5.2	38 ± 4
ALT (IU/L)	35 ± 8.3	36 ± 6.7
GGT (IU/L)	26 ± 5.6	26 ± 4
ALK (IU/L)	48 ± 8	47 ± 6.4
CRP (mg/dL)	14.1 ± 4.7	12.2 ± 4.5

Abbreviations: WBC, white blood cells; RBC, red blood cells; MCV, mean corpuscular volume; Hb, hemoglobin; IU, international units; PLT, platelets; AST, aspartate aminotransferase; ALT, alanine aminotransferase; GGT, gamma-glutamyl transferase; ALK, alkaline phosphatase; CRP, C-reactive protein.

**Table 3:** Multivariate regression analyses investigating the main covariates associated with improvements in clinical scores after fasting.

Independent Variables	Coefficient	Standard Error (SE)	*t*	*p*-Value	*r_partial_*	*r_semipartial_*
Delta BASDAI						
(Constant)	5.04					
Age	−0.05	0.03	−1.63	0.1480	−0.52	0.48
Therapy	0.58	0.50	1.15	0.2883	0.40	0.34
Gender	0.33	0.51	0.65	0.5397	0.24	0.19
BMI	−0.14	0.15	−0.95	0.3732	−0.34	0.28
Delta CRP						
(Constant)	3.98					
Age	−0.03	0.03	−0.83	0.4132	−0.15	0.14
Therapy	−0.46	0.53	−0.86	0.3942	−0.15	0.15
Gender	0.48	0.57	0.85	0.3997	0.15	0.15
BMI	−0.01	0.18	−0.043	0.9658	−0.01	0.01
Delta DAPSA						
(Constant)	12.06					
Age	−0.05	0.05	−1.10	0.2785	−0.19	0.19
Therapy	−0.66	0.78	−0.86	0.3991	−0.15	0.14
Gender	0.94	0.83	1.14	0.2627	0.20	0.19
BMI	−0.25	0.27	−0.93	0.3601	−0.16	0.16
Delta Dactylitis Severity Score						
(Constant)	−4.13					
Age	0.01	0.02	0.41	0.6845	0.07	0.07
Therapy	−0.32	0.30	−1.04	0.3063	−0.18	0.17
Gender	−0.52	0.32	−1.60	0.1186	−0.27	0.26
BMI	0.24	0.11	2.28	0.0293	0.37	0.37
Delta Leeds Enthesitis Index						
(Constant)	−1.71					
Age	0.02	0.01	1.08	0.2939	0.24	0.21
Therapy	0.57	0.26	2.22	0.0390*	0.45	0.44
Gender	−0.19	0.27	−0.72	0.4833	−0.16	0.14
BMI	0.02	0.08	0.23	0.8212	0.05	0.04

Abbreviations: BASDAI, Bath Ankylosing Spondylitis Disease Activity Index; BMI, body mass index); CRP, C-reactive protein; DAPSA, disease activity index for psoriatic arthritis.
